# Editor's Letter-Box

**Published:** 1900-06-23

**Authors:** Maria Poole


SIR,—In your issue dated June 9th, speaking of feeble-minded children under the Asylums Board being taught marketing, you express a hope that you may yet "see the children at our pauper schools going out to buy the work-house dinner." This certainly you will not see, for there is no connection between the London Poor Law schools and the workhouse—the schools are entirely separate buildings, mostly far away in the suburbs or country. But the children in the schools shopping for themselves and for their masters and matrons you could see any day. The children are encouraged and accustomed in every school to spend their own money wisely or to save it. They are sent out for errands, and in spite of constant assertions to the contrary fully understand the use and value of money. Let anyone who doubts this fact pay a visit to any London Poor Law school and try for himself.—I am, sir, yours faithfully,

MARIA POOLE.

*** The whole subject of the so-called barrack schools was threshed out recently before the Poor Law Schools Committee, with the result that the system was condemned. Of course, we are aware that the report of the committee has been very severely criticised, and that many examples may be met with of bright, intelligent, and healthy children among the pupils at these schools, but no doubt the committee paid regard rather to averages and to the general tendency of the system than to individual cases. Still it is gratifying to find that good results can be obtained even under a system which is far from being ideal, and as testing the advance which has been made of late, and as showing what is being done at the present moment to give the children of the State a proper start in life, we would draw attention to the London Poor Law Schools Exhibition which will be held in the Westminster Town Hall on July 12th, 13th, and 14th, and to the musical and other performances to be given on the same days at the Church House. On this occasion people will be able to see for themselves what is being done in the pauper schools to depauperise the rising generation.—ED. *T.H.*

